# Influence of the Ground Electrode on the Dynamics of Electrowetting

**DOI:** 10.3390/mi14020348

**Published:** 2023-01-30

**Authors:** Iftekhar Khan, Stefania Castelletto, Gary Rosengarten

**Affiliations:** 1 Future Technologies, College of VE, RMIT University, Melbourne, VIC 3000, Australia; 2 School of Engineering, RMIT University, Melbourne, VIC 3000, Australia

**Keywords:** electrowetting on dielectric, Debye double layer, liquid-liquid interface, contact-angle change

## Abstract

The ability to manipulate a liquid meniscus using electrowetting has many applications. In any electrowetting design, at least two electrodes are required: one forms the field to change the contact angle and the other functions as a ground electrode. The contribution of the ground electrode (GE) to the dynamics of electrowetting has not yet been thoroughly investigated. In this paper, we discovered that with a bare ground electrode, the contact angle of a sessile drop increases instead of decreases when a direct current (DC) voltage varying from zero to the threshold voltage is applied. This phenomenon is opposite to what occurs when the GE is coated with a dielectric, where the contact-angle change follows the Lippmann–Young equation above the threshold voltage of electrowetting. However, this behaviour is not observed with either a dielectric-coated electrode using direct current (DC) or a bare ground electrode using alternating current (AC) voltage electrowetting. This study explains this phenomenon with finite element simulation and theory. From previous research work, the ground electrode configuration is inconsistent. In some studies, the ground electrode is exposed to water; in other studies, the ground electrode is covered with dielectric. This study identified that an exposed ground electrode is not required in electrowetting. Moreover, this research work suggests that for applications where precise control of the contact angle is paramount, a dielectric-coated ground electrode should be used since it prevents the increase in the contact angle when increasing the applied potential from zero to the threshold voltage. This study also identified that contact angle hysteresis is lower with a Cytop-coated ground electrode and DC voltage than with a bare ground electrode using AC or DC voltages.

## 1. Introduction

Electrowetting is used in several applications, such as micro-drop generation, mixing and splitting [1,2], high-speed droplet actuation [3,4], chip cooling [5], drug release and clinical diagnosis [6,7], e-paper and electronic display [8,9], energy harvesting [10], solar indoor lighting [11], optics and beam steering [12,13]. In most electrowetting studies, the primary focus has been to observe the drop deformation and contact-angle change when the applied voltage is varied. At least two electrodes are required to provide the potential difference. 

Electrowetting on dielectric (EWOD) can be described using the Lippmann–Young equation, which is given by:

(1) $$\cos\theta_{R}=\cos\theta_{Y}+\frac{\varepsilon_{0}\varepsilon_{r}V^{2}}{2d\gamma_{LV}}$$

The final contact angle, $\theta_{R}$, depends on the initial contact angle, $\theta_{Y}$, the applied voltage, V, the interfacial tension between the liquid and the surrounding fluid (gas or immiscible liquid), $\gamma_{LV}$, the relative permittivity of the material, $\varepsilon_{r}$, the permittivity of free space, $\varepsilon_{0}$, and the thickness, d, of the insulating layer. 

**Figure 1:**
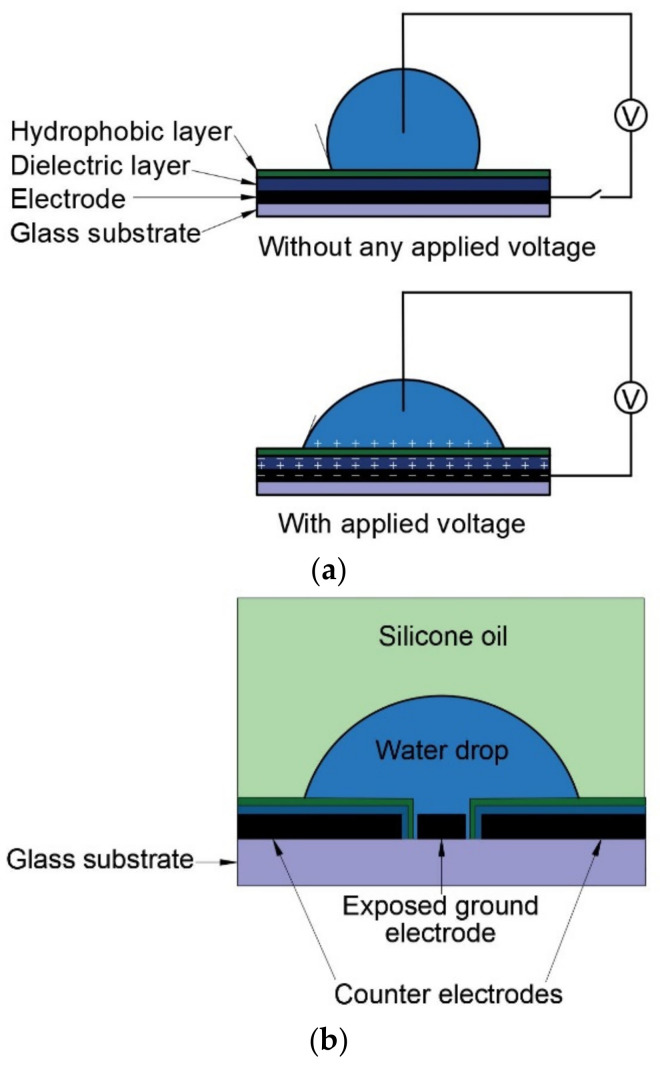

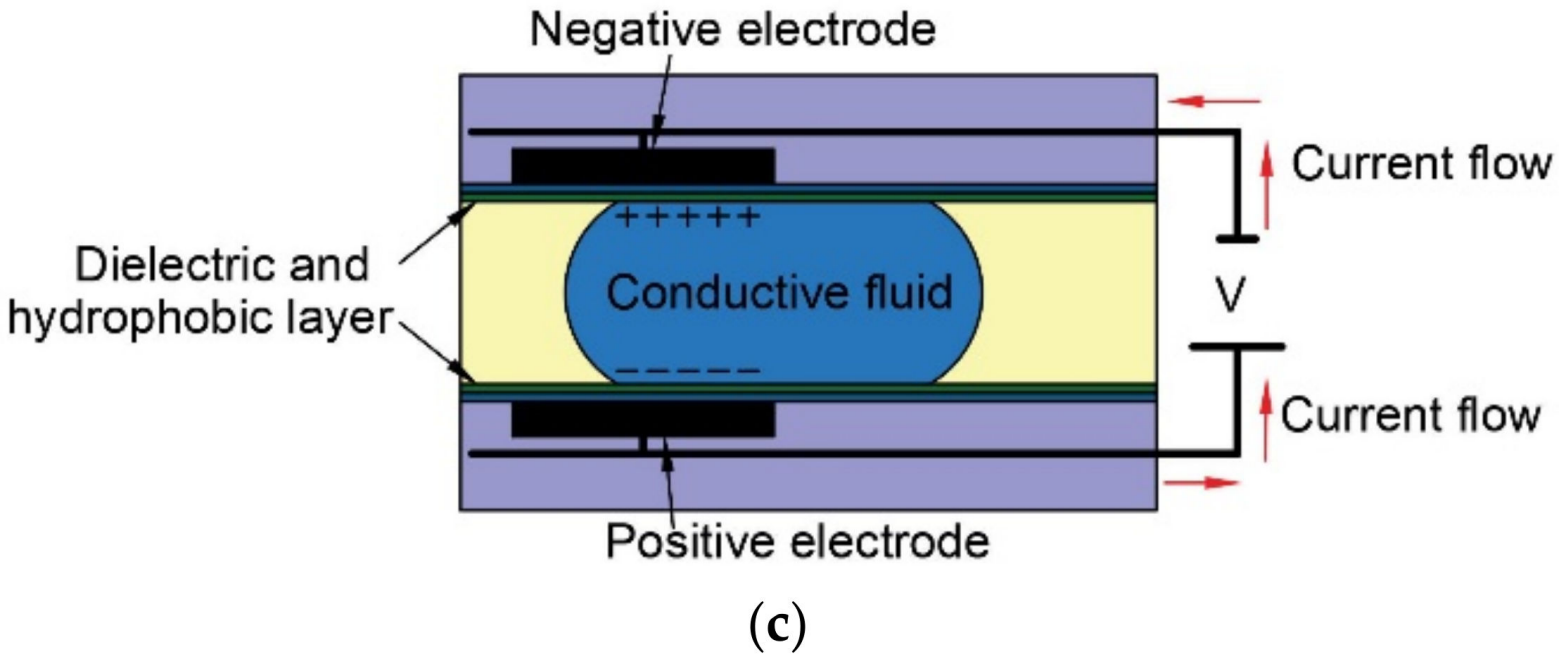
Design types and configurations in recent studies on electrowetting (**a**) sessile-drop electrowetting. Adapted from [14], (**b**) co-planar electrowetting. Adapted from [15], (**c**) parallel electrode electrowetting. Adapted from [16].

With respect to electrode position, electrowetting on dielectrics can be categorised into three different design formats: (a) sessile-drop electrowetting, (b) co-planar electrowetting, and (c) parallel-plane electrowetting, as shown in Figure 1. In sessile-drop electrowetting [14] (Figure 1a), a drop sits on a dielectric, hydrophobic layer that covers the bottom electrode. A negative potential is applied to the bottom electrode, and the positive terminal is connected to the ground electrode that is inserted from the top. In co-planar electrowetting, no ground electrode is inserted into the liquid. Instead, at least two electrodes are patterned on the bottom surface and coated with dielectric layers. With the application of a voltage, the sessile drop sitting on top of the electrode–dielectric layers deform. In a study of co-planar electrowetting [15] (Figure 1b), the ground electrode at the centre of the bottom plane was exposed to water, whereas the other electrodes were coated with dielectric and hydrophobic layers. In parallel plane electrowetting, two electrodes are positioned opposite and parallel to each other [16] (Figure 1c). From the above examples, the requirement of the ground electrode is ambiguous, as, in some studies, the ground electrode is exposed to water, and in other studies, the ground electrode is covered with dielectric.

An analysis of these designs identifies a gap in understanding how the ground electrode affects the electrohydrodynamic behaviour of the liquid in electrowetting and the differences in electrowetting performance when using a bare ground electrode or a dielectric-coated ground electrode. This study aimed to identify whether a bare ground electrode needs to be exposed to the working liquid (water) in electrowetting. This study analysed and determined how the ground electrode affects the electrohydrodynamic behaviour of the liquid during electrowetting. Furthermore, the electrowetting performance (in relation to the contact-angle change) using a bare ground electrode was compared with that using a dielectric-coated ground electrode. Finally, the study analysed and compared the performance of DC and AC electrowetting. 

To achieve this, in Section 2, we first present the theoretical model of dipole water molecule’s charge dynamics and electrical field concentration during electrowetting phenomena, followed by the experimental methods and materials used in this study in Section 3 and the experiment results in Section 4. Section 5 represents the simulation model for understanding the physics behind the experiment results. Subsequently, Section 6 provides a detailed discussion of the experimental results, co-relating them with fundamental physics and simulation outcomes. Finally, Section 7 provides a brief conclusion on the key findings of this research study. 

## 2. Theoretical Background on Dipole Water Molecule’s Charge Dynamics and Electric Field Concentration in Electrowetting

The electromechanical approach of electrowetting explains the dipole water molecule’s charge dynamics and electric field concentration in electrowetting. Several studies [17,18,19,20] have used this approach and associated equations to explain electrowetting phenomena. In this study, we also used the same approach and related equations. 

Water is predominantly used as the working fluid in electrowetting devices. The surrounding medium can be air, oil, or another immiscible electrolytic solution [21]. When an electric field is applied to a sessile drop in an electrowetting experiment, the dipole molecules of water tend to align themselves with the electric field. A torque arises on the dipole molecule, which tries to align it with the applied electric field. The torque of the dipole molecules in an electric field is defined as:

(2) τ=p×E 

where p is the dipole moment, E is the electric field, and ‘×’ denotes their cross product. The dipole molecules are randomly oriented in a dielectric liquid such as water. The dielectric polarisation is described as,

(3) $$D=\varepsilon_{0}\left(1+x_{e}\right)E=\varepsilon_{0}E+P=\varepsilon_{0}\varepsilon_{r}E=\varepsilon E$$

Here, D is the electric displacement and P is the polarisation density. Additionally, $x_{e}$ is the electric susceptibility and is defined as the tendency for a dielectric material to polarise in an applied electric field.

The polarisation density P is the vector field that defines the density of a dielectric medium’s permanent or induced dipole moment. For a unit volume dv and dipole moment dp, the polarisation density is defined as:

(4) $$P=\frac{dp}{dv}$$

The relationship between bound charge density $\rho_{b}$ and polarisation density P is:

(5) $$\rho_{b}=\nabla \cdot P$$

The polarisation density P is related to the electric field E as follows:

(6) $$P=\varepsilon_{0}x_{e}E$$

Since a dielectric liquid with an applied electric field acts similarly to a conductor [17], the resultant tangential component of the electric field is zero and expressed as:

(7) $${\left[E\cdot t\right]}_{i}^{o}=0$$

**Figure 2:**
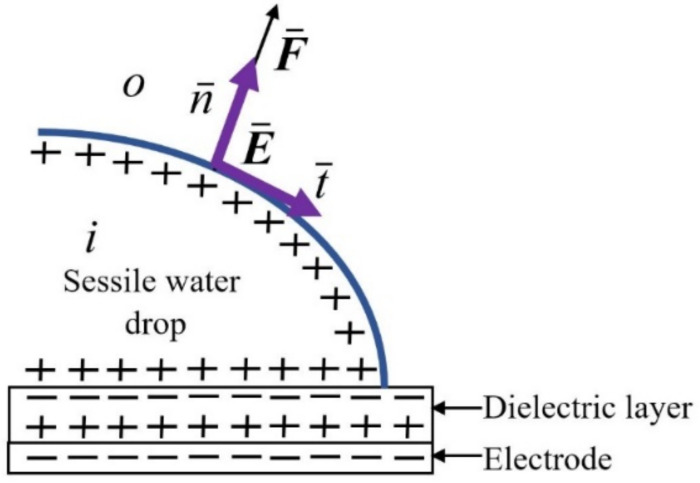
Electric field and force jump outwards, normal to the liquid surface.

Here, t denotes the unit vector tangential to the interface. The square brackets signify the jump in the interface obtained by subtracting the value of the inner phase with the notation ***i*** from that of the outer phase with the notation *o* (as shown in Figure 2). 

There is an outward jump in the electric field displacement (electric flux density) equal to the free surface charge per unit area at the interface:

(8) $${\left[\varepsilon E\cdot n\right]}_{i}^{o}=\sigma_{es}$$

Here, n denotes the unit vector perpendicular to the interface (as shown in Figure 2) and $\sigma_{es}$ is the free surface charge per unit area at the interface. The Poisson equation in a dielectric medium is given by:

(9) $$\nabla \cdot \left(\varepsilon \nabla \varphi \right)=-\rho_{e}$$

Here, $\rho_{e}$ is the volume charge density and ∇φ the scalar electrostatic potential gradient. The Maxwell electric force f, which describes electrokinetic phenomena, is represented in the Korteweg–Helmholtz force density:

(10) $$f=\rho_{e}E-\frac{1}{2}\nabla \left(\varepsilon -\rho {\left.\frac{\partial \varepsilon }{\partial \rho }\right|}_{T}\right)E\cdot E$$

The first term on the right side of Equation (10) represents the Coulomb force due to the volume charge density $\rho_{e}$. The second term on the right side of this equation has two components. The first component represents the gradient of permittivity at the interface, which occurs due to the inhomogeneity of permittivity of the two different mediums at the interface. The second component denotes the gradient of permittivity by the gradient of the density of the liquid. For an incompressible fluid, this component can be omitted from the equation.

Considering Equations (3) and (9) for an incompressible liquid, we can rewrite Equation (10) as follows:

(11) $$f=\left(\nabla \cdot D\right)E-\frac{1}{2}E\cdot E\nabla \varepsilon$$

Equation (11) can also be written as:

(12) $$f=\nabla \cdot \left[\varepsilon EE-\frac{1}{2}\varepsilon \left(E\cdot E\right)I\right]$$

Here, I denotes the second-order isotropic tensor. Equation (12) can be expressed as the divergence of a tensor:

(13) $$f=\nabla \cdot T_{M}$$

Therefore, the Maxwell stress tensor is written as:

(14) $$T_{ik}=\varepsilon_{0}\varepsilon \left(E_{i}E_{k}-\frac{1}{2}\delta_{ik}E^{2}\right)$$

where $E^{2}$ corresponds to $\left|E^{2}\right|$ and $\delta_{ik}$ is the Kronecker delta function; $\delta_{ik}=0$ if i≠k and $\delta_{ik}=0$. Here, i and k denote the x and y coordinate directions. By integrating Equation (13), we obtain the force acting on an elementary volume dV. This force is also the same as that obtained from integrating the momentum flux density or the Maxwell stress tensor on the surface of the volume dV. Using the Gauss divergence theorem, we can identify the total force on the body:

(15) $$F=\int_{V}fdV=\int_{V}\nabla \cdot T_{M}dV=\int_{S}n\cdot T_{M}dA$$

The applied electric potential generates this body force F, which in turn deforms the liquid and changes the contact angle, and the process called electrowetting occurs. In this study, the equations above were used to model the electric field in a sessile drop to predict the force.

## 3. Materials and Methods

**Figure 3:**
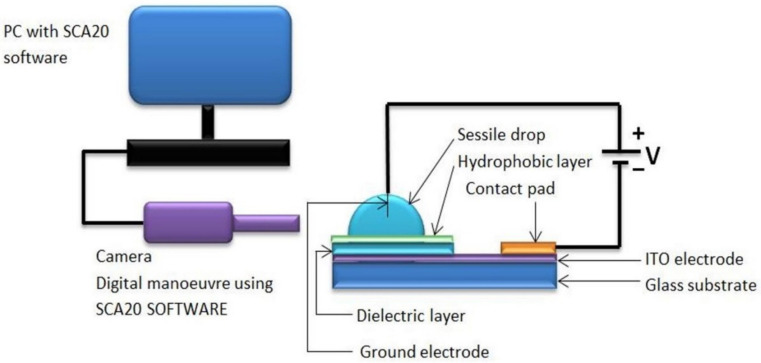
Experimental setup for sessile-drop electrowetting.

To answer the questions posed in the introduction section, sessile-drop electrowetting experiments were conducted. Deionised water was used as the liquid for the sessile drop with an electrical conductivity of 10^−6^ S/cm. As shown in Figure 3, a sessile drop was placed on top of the hydrophobic–dielectric layer. These layers are coated on a transparent ITO (indium tin oxide) electrode layer on top of the glass substrate. In most of the sessile-drop electrowetting studies, such as in [22,23], the bottom planar dielectric-coated ITO electrode is used as the working electrode, and the wire which is inserted into the sessile drop is used as the ground electrode. We followed the same method as used in standard sessile-drop electrowetting studies. A conductive contact pad was used to connect the ITO with the negative terminal of the power supply. A thin ground wire (100 µm) was inserted into the liquid, and the other end of the wire was connected to the positive terminal of the power supply. Electrowetting occurred when a voltage potential was applied to the circuit, and the sessile drop spread on the surface. A goniometer’s camera captured the image of the sessile drop, and the SCA20 software derived the contact angle. 

In sessile-drop electrowetting, an oil ambient can avoid any effect of evaporation and reduce contact angle hysteresis. However, there is a problem with using an oil ambient for the sessile-drop electrowetting test. During the wetting and de-wetting process of an oil ambient sessile drop, a thin oil layer can be entrapped between the sessile drop and dielectric surface. This oil film can be µm thick and can create contact line instability. When electrowetting occurs, this thin entrapped oil film can break up periodically and form small oil droplets. This contact line instability and the formation of small oil droplets is termed spinodal de-wetting. Several studies [8,24] have noted the spinodal de-wetting problem when oil is used as the second medium or as an ambient medium surrounding water. This problem can be avoided when air is used as the ambient medium. With air as the surrounding medium, the water drop is directly on the hydrophobic–dielectric-coated electrode surface, and because of this reason, this study avoided the use of oil as the ambient medium surrounding the water. Furthermore, the experiments were done rapidly to minimise evaporation. 

To fabricate the sessile-drop electrowetting surface, a 100 nm ITO layer was first deposited on top of a glass substrate using electron-beam deposition. After this, the ITO layer was annealed at 450 °C for four hours to improve its adhesion to the glass surface and increase its electrical conductivity. As a dielectric material, a 100 nm thick Al_2_O_3_ layer was deposited by atomic layer deposition (ALD) on top of the ITO layer. In addition, some studies [14,25] have suggested that a two-layer dielectric-hydrophobic material can reduce defects and help to prevent dielectric breakdown. They have also stated that an inorganic first layer with an organic-hydrophobic second layer increases the breakdown voltage limit. In recent studies [26,27], Cytop (an organic hydrophobic material) has shown better performance in electrowetting because of its high breakdown voltage compared to other hydrophobic-dielectric materials. Therefore, Cytop was chosen as the hydrophobic material to be deposited on the Al_2_O_3_ dielectric layer in this study.

Additionally, to improve the adhesion of Cytop to the Al_2_O_3_ layer, an adhesion promoter solution was used. This solution was prepared by mixing 0.1% amino silane agent to a mixture of ethanol (95%) and deionised (DI) water (5%). After the adhesion promoter was spin-coated, a 4% Cytop 809 solution was spin-coated and then baked to produce the 100 nm thick Cytop layer.

The experimental study conducted sessile-drop electrowetting with a bare ground wire and a Cytop-coated ground wire. For the latter, the same procedure was followed to coat a ground wire with Cytop. In this study, AC and DC voltage were used separately to change the contact angle in sessile-drop electrowetting. Only the positive part of the sinusoidal curve was used for the positive AC voltage electrowetting experiment. Only the negative part of the sinusoidal curve was used for the negative AC voltage electrowetting experiment. Additionally, a high frequency was used to avoid vibration of the sessile drop [23,28]. A 10 kHz AC voltage was generated using a function generator and a custom-made high-frequency transformer. The function generator supplied high-frequency positive or negative waveform AC voltage, and the transformer amplified the output. 

## 4. Results

Several experiments were conducted to investigate the ground electrode’s contribution to the electrowetting phenomenon. First, this study investigated how the contact-angle changed in sessile-drop electrowetting with a DC voltage supply and a bare ground electrode compared to a Cytop-coated ground electrode. The contact-angle change in sessile-drop electrowetting with both DC and AC voltage was also examined, with the main aim being to determine whether the contact-angle change in electrowetting differed with a change in the type of applied voltage.

**Figure 4:**
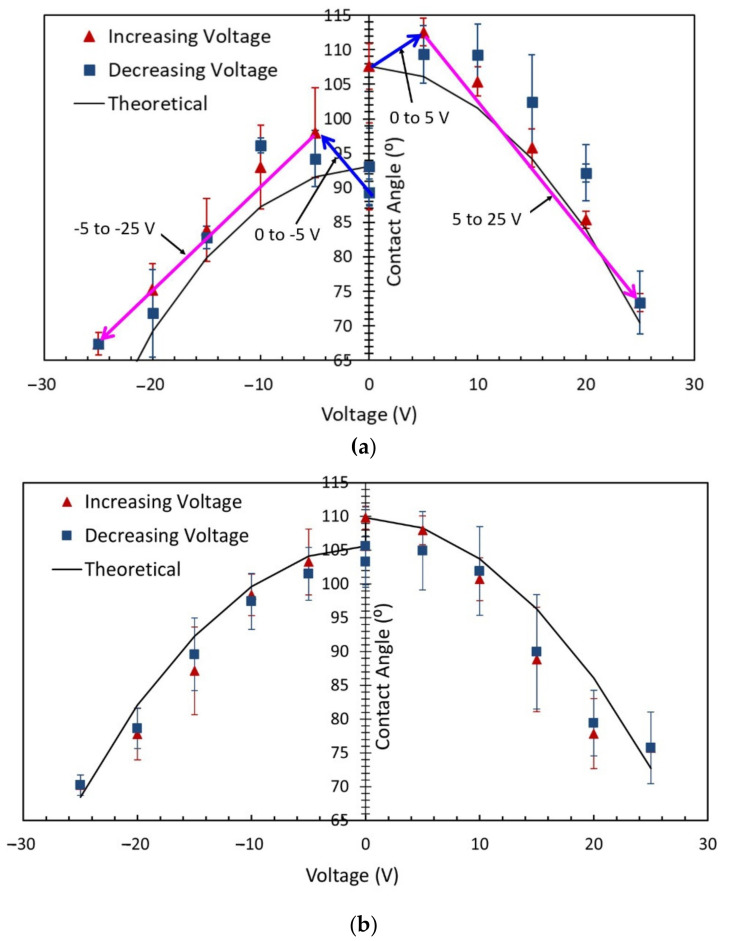

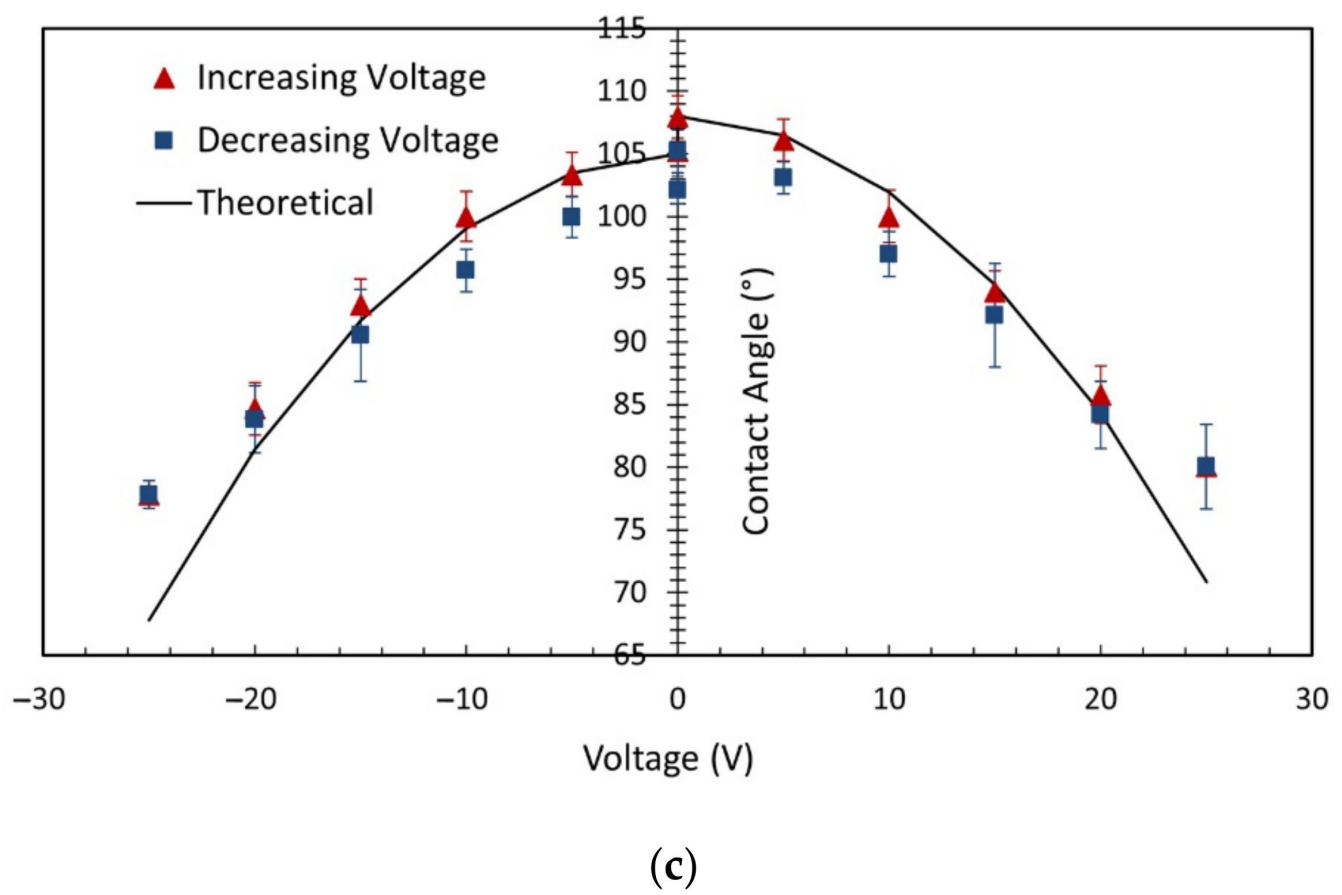
Sessile-drop electrowetting with (**a**) DC voltage and the bare ground electrode, (**b**) DC voltage and Cytop-coated ground electrode, and (**c**) AC voltage and the bare ground electrode.

Figure 4a shows the contact angle in sessile-drop electrowetting with a bare ground electrode and a DC power supply. The graph presents the contact angles in both positive and negative potential in forward electrowetting (increasing voltage) and backward electrowetting (decreasing voltage). Each data point in the graph represents the average of repeated data sets, and error bars were calculated using the mean standard deviation for each measurement. There are two error bars at each data point, one for increasing voltage (red colour) and another for decreasing voltage (dark blue colour). Additionally, the theoretical contact-angle curve presented in the graph was calculated using the Lippmann–Young Equation (1). A similar procedure was followed in Figure 4b,c. As seen in Figure 4, an interesting phenomenon was observed during this experiment, which, to the author’s knowledge, has not so far been noted in any previous research studies. According to the Lippmann–Young Equation (1), in sessile-drop electrowetting, the liquid drop spreads on the dielectric layer’s surface, and the contact angle gradually decreases with increasing voltage. Experimentally, this change in contact angle usually occurs beyond the threshold voltage of electrowetting. The value of the threshold voltage depends on the properties of the dielectric material, such as the dielectric constant and the thickness of the dielectric layer. As shown in Figure 4a, in this study, as the voltage gradually increased from 0 V to 5 V, the contact angle also gradually increased. The error bar (mean standard deviation) at 5 V was 3.25°. During the experiment, the voltage was increased at 1 V increments. However, the contact angle measurement was taken at 5 V to complete the experiment quickly and avoid the drop’s evaporation. According to this equation, the contact angle should decrease with increasing voltage, as was observed from 5 V onwards. As shown in Figure 4a, 5 V can be considered the threshold voltage for these electrowetting experiments, since electrowetting phenomena were observed to occur beyond this value. From 10 V onwards, the contact angle was in close agreement with the theoretical contact-angle value, and it reached 73° (average) at 25 V (Figure 4a).

Similar behaviour was observed when the voltage was gradually reversed (as shown in Figure 4a). The contact angle at 25 V was then initially 73° (average) at 25 V. It returned to 109° (average) at 5 V and then sharply dropped to 93° (average) at 0 V. As revealed in Figure 4a, the contact angles of the sessile drop were different at the beginning of the experiment and at the end when the voltage returned to 0 V. This may be because the sessile drop evaporated during the forward and backward electrowetting in the experiment since evaporation is known to cause a reduction in the contact angle [29,30]. According to the Lippmann–Young theory, let us consider the contact angle of 93° at 0 V during the reversed electrowetting experiment. The contact angle should gradually return to this value from 73°, not increase to 109° at 5 V, and then drop down to 93°. Similar phenomena were also observed when the negative potential was applied to the bottom ITO electrode, gradually increasing, and then reversed. The negative potential experiment was started after completing the positive potential forward and reverse electrowetting experiment. Because of the time delay, there was evaporation of the water droplet, and the contact angle was subsequently different at 0 V. The experimental data shows that an extra upward force on the droplet from the ground electrode pulled the sessile drop upward (this is not considered in the Lippmann–Young equation). Figure 4a also indicates a hysteresis of 6° between 10 V and 20 V.

Figure 4b shows the contact-angle change in sessile-drop electrowetting with a Cytop-coated ground electrode and DC voltage supply (both the positive and negative potential). When this electrode was used, no unusual behaviour was observed during forward and reverse voltage electrowetting in the range of 0 to 5 V, as shown in Figure 4a. From Figure 4b, it can be noted that the threshold voltage of this experiment was also 5 V, beyond which the contact angle decreased sharply with increasing voltage. From 0 V to 5 V, there was a small change in contact angle. Furthermore, during the reverse voltage electrowetting, no upward or downward change in contact angle was observed as the voltage moved from the threshold to 0 V and vice versa, as seen in Figure 4a. Figure 4b also reveals that the hysteresis was negligible from the threshold voltage of 5 V to 20 V. The contact angle was reduced by 4° during reverse electrowetting when the voltage returned to 0 V. This may be due to the evaporation of the sessile drop during the experiment. Similar contact-angle changes were also observed with the Cytop-coated ground electrode when the electrowetting experiment was conducted with a negative DC potential at the bottom ITO electrode. 

The sessile-drop AC electrowetting experiment used a bare ground electrode to investigate how the contact-angle change differed between AC and DC actuation. As shown in Figure 4c, with AC voltage, a slight decrease in contact angle from 0 to 5 V was detected, and the contact-angle change was more evident when the voltage was increased to 5 V or higher. From these experimental data, 5 V can be marked as the threshold voltage of electrowetting. This value is consistent with the previous electrowetting experiments in Figure 4a,b. In sessile-drop AC electrowetting experiments with a bare ground electrode, no similar change in contact angle was seen as the voltage rose from zero to the threshold voltage, as observed before with the bare ground electrode and DC voltage supply. A similar statement can be made for the reverse electrowetting experiments with a bare ground electrode and AC voltage supply (Figure 4c). Finally, it is evident from the experimental results (Figure 4c) that the contact angle in sessile-drop electrowetting followed a trend similar to the theoretical curve. Figure 4c also indicates that a contact-angle hysteresis of an average of 3° was observed with the bare ground electrode and AC voltage supply. In comparing Figure 4a–c, it can be noted that the hysteresis of the contact angle was the least with the Cytop-coated ground electrode in sessile-drop DC electrowetting. Less hysteresis was witnessed in AC electrowetting than in DC electrowetting with the bare ground electrode. A similar contact-angle change was also observed when the electrowetting experiment was conducted with negative potential at the bottom ITO electrode.

According to the Lippmann–Young equation, the electric potential at the triple-phase contact line plays a vital role in electrowetting, irrespective of the polarity of the applied voltage. Figure 4 shows that similar electrowetting results were obtained from both the positive and negative potential. This outcome agrees with the studies of [8,31], which state that the contact-angle change in electrowetting is independent of the polarity of the applied potential. If there is any difference in contact-angle change with the applied potential, that may be related to the preferential charge absorption of the dielectric material. As noted in the studies [32,33], the dependence of the extent of wetting on the electrode polarity is most likely related to the preferential absorption of hydroxide ions (OH−).

**Figure 5:**
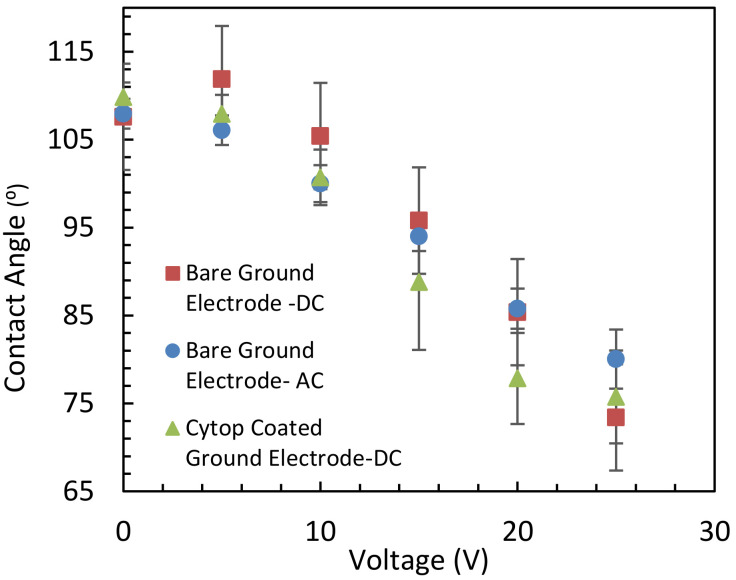
Comparison of sessile-drop electrowetting with a bare ground electrode and forward DC voltage, a bare ground electrode and forward AC voltage, and a Cytop-coated ground electrode and forward DC voltage.

Figure 5 compares the contact-angle change in sessile-drop electrowetting with a bare ground electrode and forward DC voltage, a bare ground electrode and forward AC voltage, and a Cytop-coated ground electrode and forward DC voltage. As observed, the contact angle increased as the voltage increased from zero to the threshold voltage of 5 V in DC electrowetting with a bare ground electrode, whereas the contact angle remained the same or slightly decreased with the Cytop-coated ground electrode and DC voltage, and with the bare ground electrode and AC voltage. Furthermore, the contact-angle change was less in AC electrowetting (average of 27°) than in DC electrowetting (where the average contact-angle change was 34° with both bare and Cytop-coated ground electrodes).

**Figure 6:**
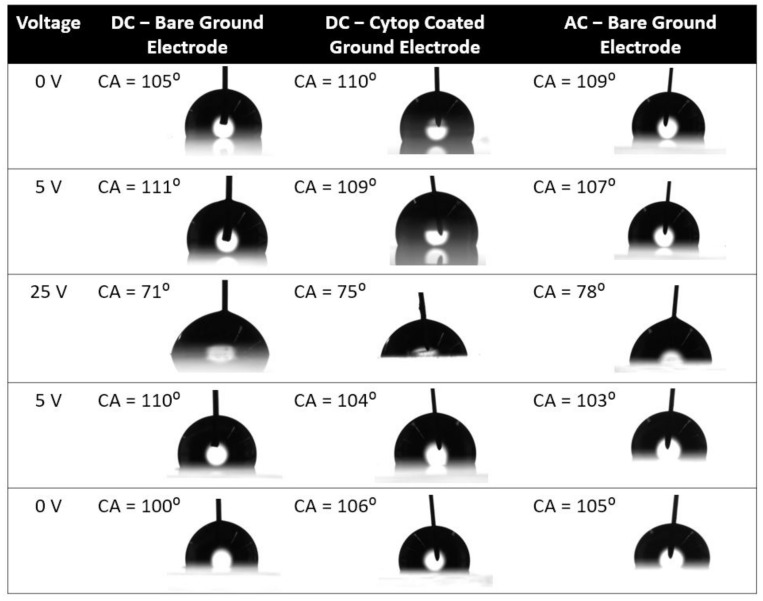
Contact-angle (CA) of the sessile drop in electrowetting with DC-bare ground electrode, DC-Cytop-coated ground electrode and AC-bare ground electrode. Time increases from top to bottom with the voltage increasing and then decreasing.

Figure 6 presents images of the sessile drop in the forward- and reverse-voltage electrowetting experiments with a bare ground electrode and DC voltage, a Cytop-coated ground electrode and DC voltage, and a bare ground electrode and AC voltage. As reported in the Figure 6, in the DC electrowetting experiment with the bare ground electrode, the contact angle increased from 105° at 0 V to 111° at 5 V, whereas in the other electrowetting experiments, the contact angle gradually decreased. Similar phenomena were observed during the reverse-voltage electrowetting experiments. 

In the following sections, the simulation study and theoretical framework are presented to aid the understanding of the experimental results. 

**Figure 7:**
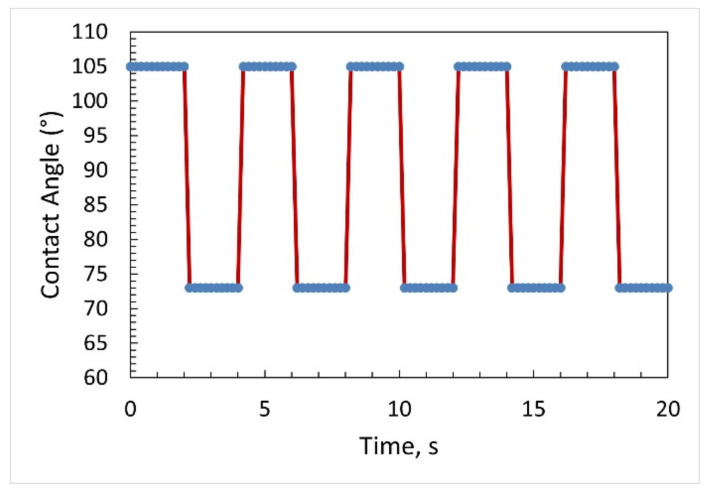
The contact-angle change as a function of time for several voltage cycles from 0 to 25 V DC in a bare ground sessile-drop electrowetting experiment.

To test the switching speed of the sessile drop, an electrowetting experiment was conducted by repeatedly switching the applied voltage from 0 V to 25 V. The voltage was changed at 2 s intervals. Figure 7 shows the contact-angle change as a function of time for several voltage cycles from 0 to 25 V DC in a bare ground sessile-drop electrowetting experiment. From the graph, it is evident that the sessile drop response was fast. 

## 5. Simulation of the Electric Field

Simulations were carried out to understand the experimental results presented in the previous section. The primary objective of these simulations was to determine how the electric potential and electromotive force develop in a sessile drop with a bare ground electrode and a dielectric-coated ground electrode when the drop acts as a leaky dielectric [34,35] or as a pure conductor. The commercial software COMSOL Multiphysics 5.2 was used, and in the electric field simulations water is assumed to be a continuum. The simulation does not consider the molecular dynamics of the dipole molecules of water and the formation of a Debye double layer. Therefore, they represent only the macroscopic electric field simulation results. In the simulation model, the laminar two-phase flow and level set method were used to model the liquid’s interface shape. The software’s electrostatic module was used to calculate the associated electric field with the applied electric potential. 

**Figure 8:**
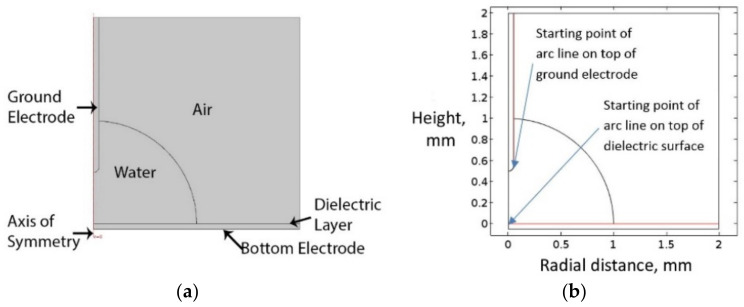
(**a**) Boundary conditions of the simulation study, (**b**) coordinate system for plotting the electric field magnitude and integration calculations of the resultant force on the dielectric and ground electrode surfaces.

Figure 8a presents the boundary conditions of the simulation model. An axisymmetric geometry was used in the simulation to represent water, air, the ground electrode, the bottom electrode, and the dielectric material. Figure 8b shows the coordinate system for plotting the electric field magnitude and integration calculations of the resultant force on the dielectric and ground electrode surfaces.

### 5.1. Bare Ground Electrode Exposed to a Sessile Water Drop

**Figure 9:**
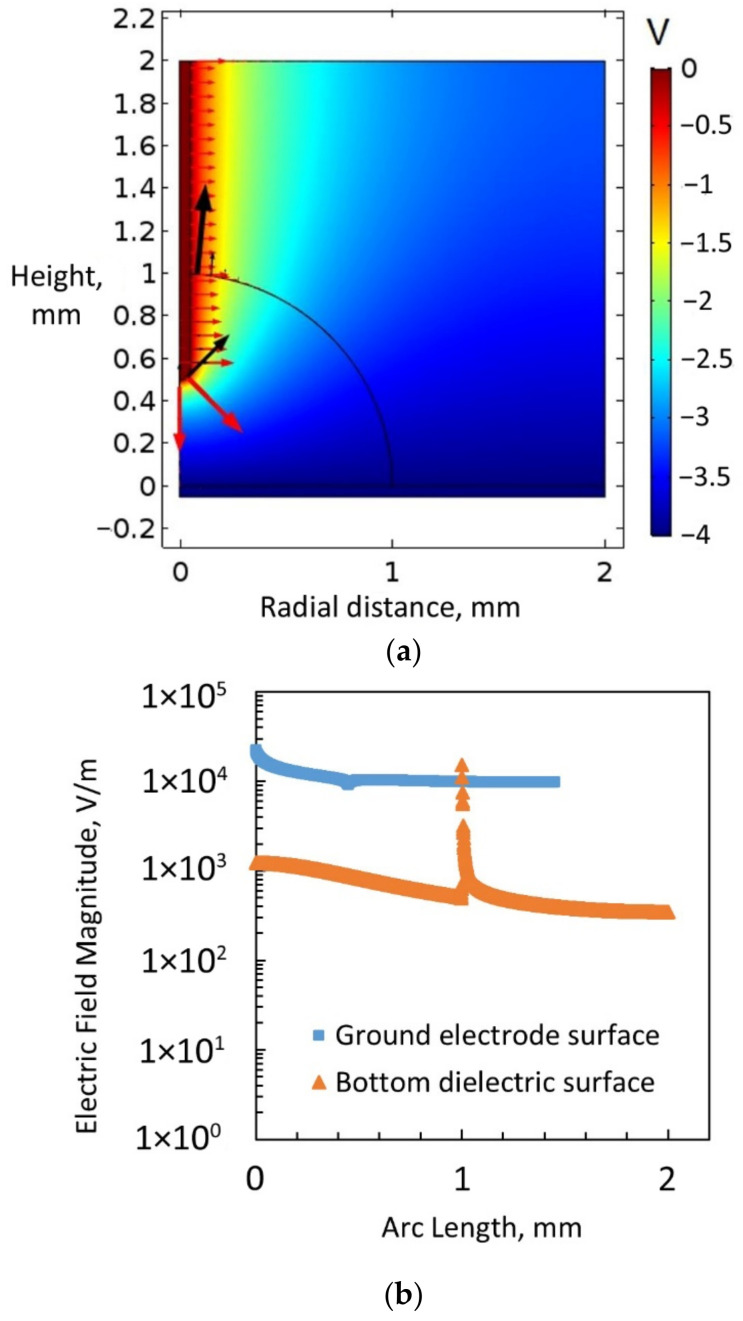
(**a**) Electric field simulation considering water to be a leaky dielectric, with a bare ground electrode exposed to a sessile water drop and 4 V applied to the bottom electrode covered with a dielectric. (**b**) The electric field magnitude on the ground electrode surface and the bottom dielectric surface, considering water to be a leaky dielectric (4 V applied to the bottom electrode).

**Figure 10:**
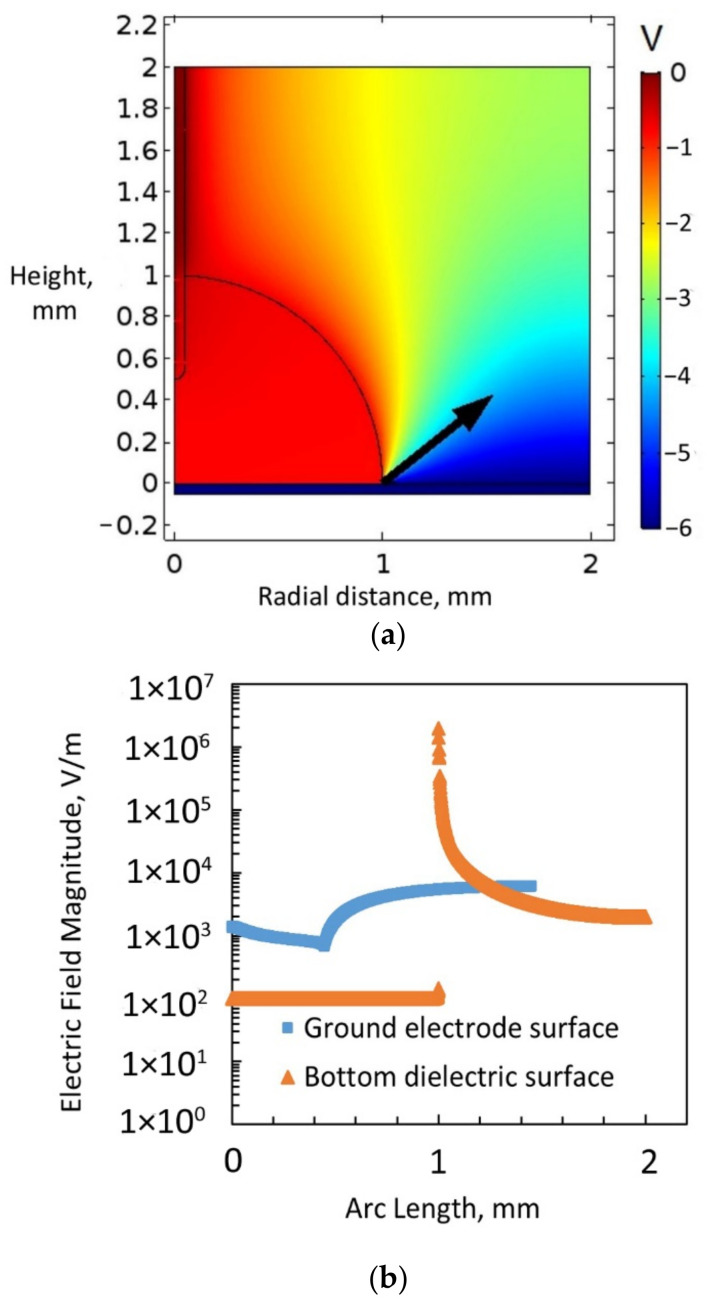
(**a**) Electric field simulation considering water to be a conducting liquid, with a bare ground electrode exposed to a sessile water drop and 6 V applied to the bottom electrode covered with a dielectric. (**b**) Electric field magnitude on the ground electrode surface and the bottom dielectric surface, considering water to be a conducting liquid (6 V applied to the bottom electrode).

**Figure 11:**
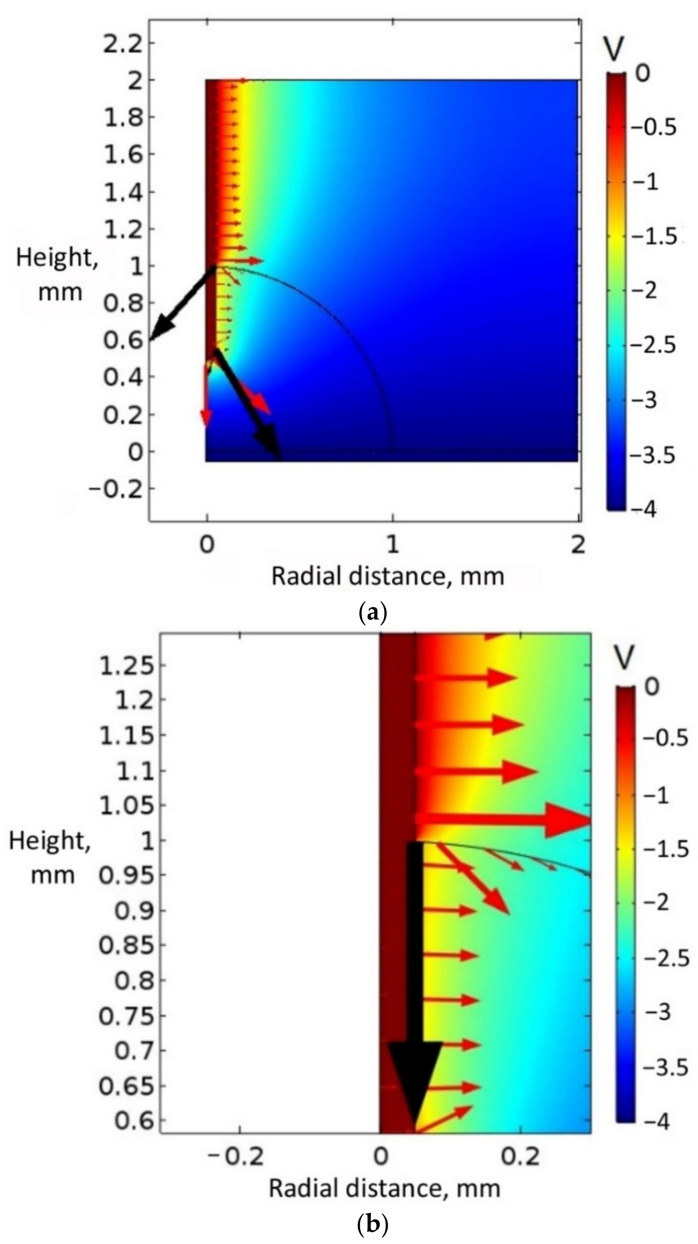

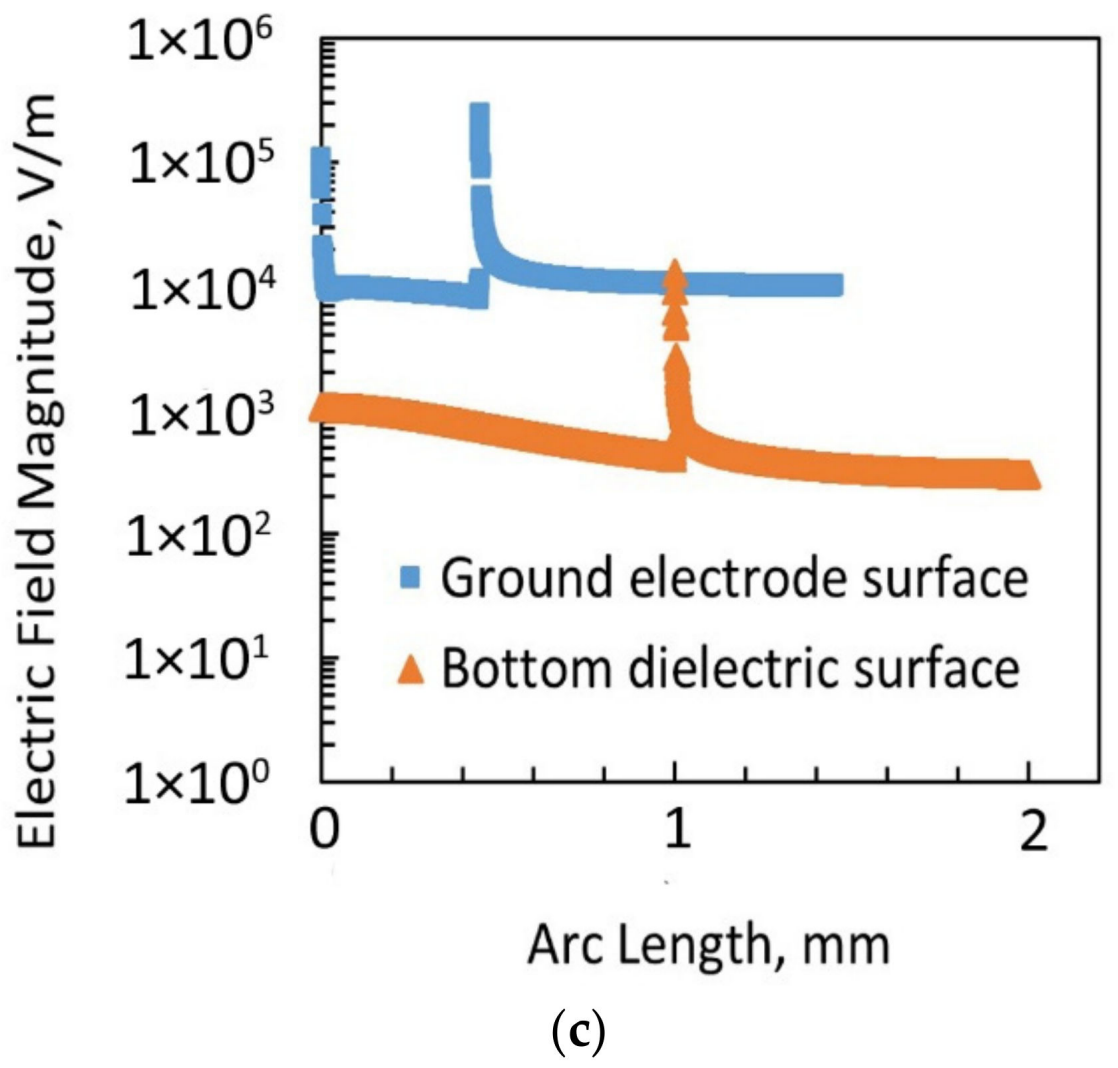
(**a**) Electric field simulation considering water to be a leaky dielectric and a dielectric-coated ground electrode exposed to a sessile water drop, with 4 V applied to the bottom electrode covered with a dielectric. (**b**) Magnified view of the simulation result representing the vertical force component (the black arrow line) on the ground electrode. (**c**) Electric field magnitude on the dielectric-coated ground electrode surface and bottom dielectric surface.

**Figure 12:**
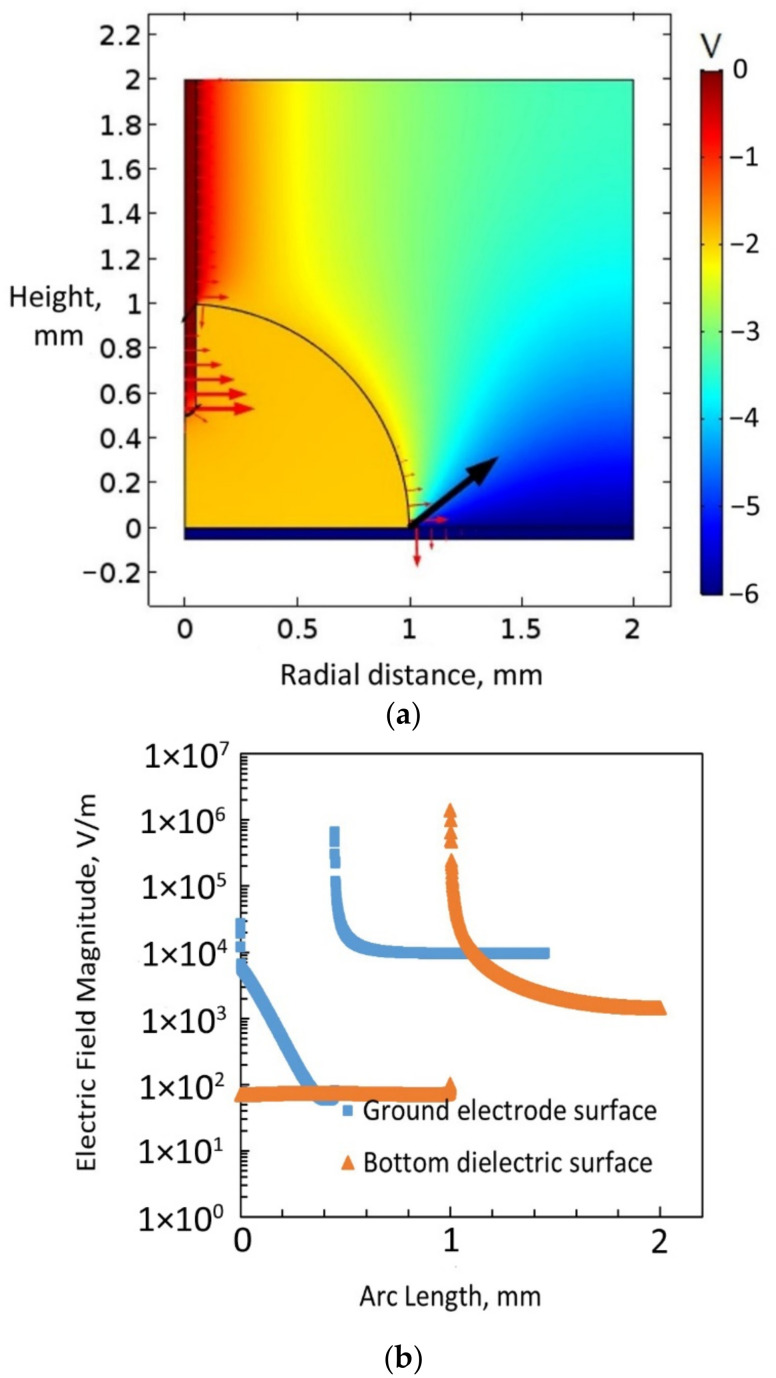
(**a**) Electric field simulation with water considered to be a conducting liquid, a dielectric-coated ground electrode inserted into a sessile water drop, and 6 V applied to the bottom electrode covered with a dielectric. (**b**) Electric field magnitude on the dielectric-coated ground electrode surface and bottom dielectric surface.

To determine how the electric potential develops when the sessile drop acts as a leaky dielectric and when it acts as a pure conductor, two separate simulations were conducted. In both simulations, the parameters were the same except for the permittivity of water. In the first simulation, the value of the relative dielectric permittivity of water was set as 80 [36]. In this simulation, the thin permittivity gap (TPG) boundary condition was defined only for the bottom electrode. The TPG boundary layer was defined as a dielectric layer with a dielectric constant of 3.5 (equivalent dielectric constant of Al_2_O_3_ and Cytop layer together as fabricated) and dielectric thickness of 200 nm. Figure 9a, Figure 10a, Figure 11a,b, and Figure 12a show the surface plot of electric potential (V), electric field lines (red arrow lines), and resultant force lines (black arrow lines) as according to Equation (15). Figure 9 illustrates that with a relative permittivity of 80, water acted as a leaky dielectric using an applied voltage of 4 V. This condition represents voltage below the threshold when the dielectric molecules of water gradually align themselves to the electric field. Figure 9a shows a voltage gradient across the water drop due to the drop’s leaky-dielectric behaviour. In this scenario, the macroscopic electric field forces were evident on the surface of the ground electrode. There was a concentration of upward force component at the triple-phase contact line of the sessile drop on the ground electrode surface. Figure 9b shows the electric field magnitude on the ground electrode surface and the bottom dielectric surface when water was a leaky dielectric. The electric field magnitude was higher on the ground electrode surface than on the bottom dielectric surface, except at the triple-phase contact point on this layer. There was an electric field concentration on top of the ground electrode at the triple-phase contact point. As seen, the electric field magnitude was maximum at the starting point of the arc line on top of the ground electrode. However, the net force on the water found by integrating the electromotive force along the ground surface electrode surface (2.1 × 10^6^ N/m^2^) was higher than that on the bottom dielectric surface (1.3 × 10^4^ N/m^2^).

In the second scenario with a bare ground electrode, the simulation considered water to be a conductive liquid (with dissolved salt, for example) with a very high relative permittivity of 1 × 10^5^. A potential of 6 V was applied to the bottom electrode. This condition represents a scenario beyond the threshold voltage of this electrowetting simulation, considering the threshold voltage was 5 V. Figure 10a displays the result of this simulation. As expected, there was no voltage drop through the water and thus no electric field in the water drop. The electric field lines emanated from the surface of the sessile drop. Thus, the electric field was concentrated at the triple contact point, and a higher electric field force was observed at the triple contact point, as indicated by the black arrows. Hence, this simulation correctly described the behaviour of the sessile drop in electrowetting beyond the threshold voltage because, beyond the threshold voltage, the dipole molecules aligned themselves with the electric field, and the liquid acts as a conductor. The forces were then sufficient to cause the observed electrowetting phenomenon. Figure 10b shows the electric field magnitude on the ground electrode surface and the bottom dielectric surface when water was a conductive liquid. The electric field magnitude over the ground electrode surface was lower than that at the triple contact point on the bottom dielectric layer. Additionally, according to the simulation, the electromotive force integration over the arc line on the bottom dielectric surface was considerably higher (2.2 × 10^8^ N/m^2^) than that over the arc line on the ground electrode surface (119 N/m^2^). Therefore, the simulation showed that when water acted as a conductive layer, a larger resultant force acted on the bottom dielectric layer, which spread the liquid droplet over the surface of this layer.

### 5.2. Dielectric-Coated Ground Electrode

To compare and understand the experimental results, simulations were conducted with a dielectric-coated ground electrode. In this set of simulations, the TPG boundary layer was defined both on top of the bottom electrode and on top of the ground electrode. The TPG boundary layers were defined as a dielectric layer with a dielectric constant of 3.5 and a dielectric thickness of 200 nm. Figure 11a presents the results of the simulation with 4 V applied to the bottom electrode and water considered to be a leaky dielectric (with an electric permittivity of 80). These parameters represent a scenario of electrowetting below the threshold voltage. Figure 11b shows a magnified image of the vertical-force component direction at the sessile-drop–ground-electrode interface. As shown in both these figures, the electric field force on the liquid-dielectric interface pushed the sessile drop downward along the dielectric-coated ground electrode surface.

Figure 11c shows the electric field magnitude on the dielectric-coated ground electrode surface and the bottom dielectric surface. As according to the simulation results in Figure 11, the electric field magnitude and the electromotive force were both higher on the dielectric-coated ground electrode surface (3.5 × 10^5^ N/m^2^) than on the bottom dielectric surface (9620 N/m^2^) when water was a leaky dielectric, and 4 V was applied to the bottom electrode. However, the macroscopic electric field simulation of Figure 11c showed that the vertical-electromotive-force component (Fz) on the surface of the dielectric coated ground acted downward compared to upward, which was the case with a bare ground electrode. Figure 12 displays the result of the simulation with a dielectric-coated ground electrode when water was a conducting liquid with a relative permittivity of 1 × 10^5^. A 6 V was applied to the bottom electrode, representing a scenario of electrowetting beyond the threshold voltage. As seen in the figure, there were voltage drops both at the bottom dielectric surface and the dielectric-coated ground electrode surface, but no voltage gradient through the water droplet. Additionally, it can be noted that the electric field magnitude was high at the triple-phase contact point on top of the bottom dielectric layer. The simulation identified that when water was a conducting liquid, the electromotive force integration on the bottom dielectric surface was considerably higher (1.18 × 10^8^ N/m^2^) than that on the dielectric-coated ground electrode surface (2.37 × 10^5^ N/m^2^). 

If water was considered as charged ions, it behaved similar to a pure conductor, showing very little or no leaky dielectric behaviour, as there were distinct differences between leaky dielectric liquid and ionic liquid [37]. The simulation with charged ions showed results as in Figure 10 and Figure 12 but did not show results as in Figure 9 and Figure 11. A detailed simulation with ionic liquid may require electrochemistry [37] and molecular dynamic simulation [38]. 

## 6. Discussion

### 6.1. Debye Double Formation and Contact-Angle Change in DC Electrowetting with a Bare Ground Electrode

In electrowetting, when a voltage is applied to a water drop, a local electromotive force develops, which creates a thin polarised layer of opposite ions on top of the ground electrode. This layer is known as the Debye double-layer. The surface charging and classical theory of Debye double layer formation on the metal surface can be found in Chang and Yeo [39]. Due to the leaky dielectric behaviour of water, a Debye double-layer quickly forms on the metal surface of the bare ground electrode as the voltage increases from zero to the threshold voltage. Because of this, a charge concentration develops at the triple-phase contact line on the surface of the bare ground electrode, which in turn creates an electric field concentration and hence an electric field force that causes the water on the bare ground electrode to rise. 

**Figure 13:**
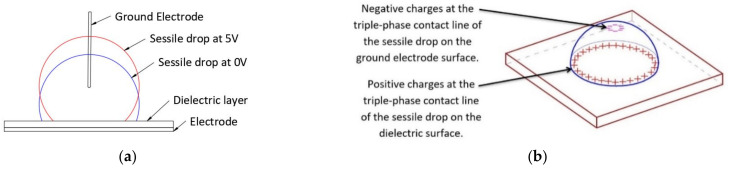
(**a**) Increase in contact angle of the sessile drop in the range of 0–5 V. (**b**) Dipole charges at the contact line of the sessile drop (when water acts as a conducting liquid).

Figure 13a presents the schematic of the increase in contact angle of the sessile drop in the range of 0–5 V. As shown in the figure, from the 0 to 5 V range, the sessile drop rises upon the ground electrode and the contact angle increases on top of the bottom dielectric-electrode-coated surface. 

**Figure 14:**
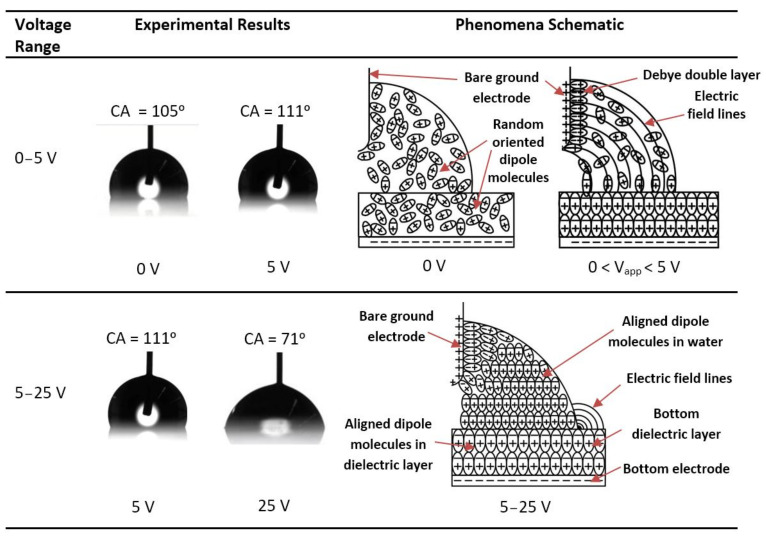
Experimental images and schematics describing the physics of sessile-drop electrowetting with the bare ground electrode and increasing DC voltage.

Figure 14 presents the sessile drop images at 0 V and 5 V. It is evident from the sessile drop images that the contact angle increased instead of decreasing for the voltage increase from zero to 5 V. Figure 14 also shows the schematic of the Debye double-layer formation on the bare ground electrode, which accounts for the contact angle increase in that voltage range. 

Additionally, in this voltage range, the Debye double-layer continues to form on the dielectric layer [40,41]. Because of this, the electric field force at the triple contact line on the ground electrode is stronger than that on top of the dielectric layer on the ground electrode. Even though the simulation mentioned previously did not account for Debye double-layer formation, the electric field propagated through the sessile drop caused a potential gradient through the liquid. A macroscopic electromotive force concentration developed on the surface of the ground electrode (Figure 9). The associated electric field force created a resultant upward force along the surface of the ground electrode in the voltage range of zero to the threshold voltage. This study suggests that the upward force along with the Debye double-layer electromotive force (not simulated here), lifts the drop, increasing the drop’s contact angle on the dielectric layer, as observed experimentally.

As the voltage increases above 5 V, dipole molecules align themselves with the applied electric field. As a result, a strong electromotive force ***F*** is generated at the triple-phase contact point on the bottom dielectric surface [17]; see Equation (15). A Debye double-layer also exists on top of the ground electrode. As shown in Figure 13b, more charge accumulates at the triple contact line on top of the dielectric material than on the triple contact line of the ground electrode, due to the contact area of the liquid on the dielectric material being larger than that on the ground wire. Hence, a higher force is exerted at the triple contact line on the dielectric material than that at the triple contact line on the ground electrode, and the liquid drop spreads over the dielectric layer surface. This state of the liquid drop is characterised by a voltage equal to or more than the threshold voltage of electrowetting. During this time, water acts as a pure electric conductor. As shown in Figure 14, the dipole molecules are aligned, and an electric field concentration develops at the bottom triple-phase contact point. The simulations also validate this assumption, as with high-electric permittivity, water acts as an electric conductor, and the electric field force is concentrated at the triple contact point at the bottom dielectric-electrode layer, as shown in Figure 10a. In the electrowetting experiments conducted in this study, the threshold voltage of electrowetting was 5 V.

Kang [17] suggested that the sessile drop acts as a pure conductor, and the electric field jumps emanate from the drop’s surface. However, the present study indicates that Kang’s assumption of water being a purely conductive liquid is not satisfied during the process of the water drop transforming from a leaky to a pure dielectric. This study postulates that all the molecules of the water drop align themselves with the electric field at the threshold voltage of electrowetting. Historically, the threshold voltage has been defined as the voltage at which the sessile drop in electrowetting experiments begins to spread on the dielectric surface. From zero to the threshold voltage, the polar molecules gradually align themselves with the electric field, the electric resistance decreases, and the liquid acts as an electric conductor. 

### 6.2. Contact-Angle Change in DC Electrowetting with a Cytop-Coated Ground Electrode

**Figure 15:**
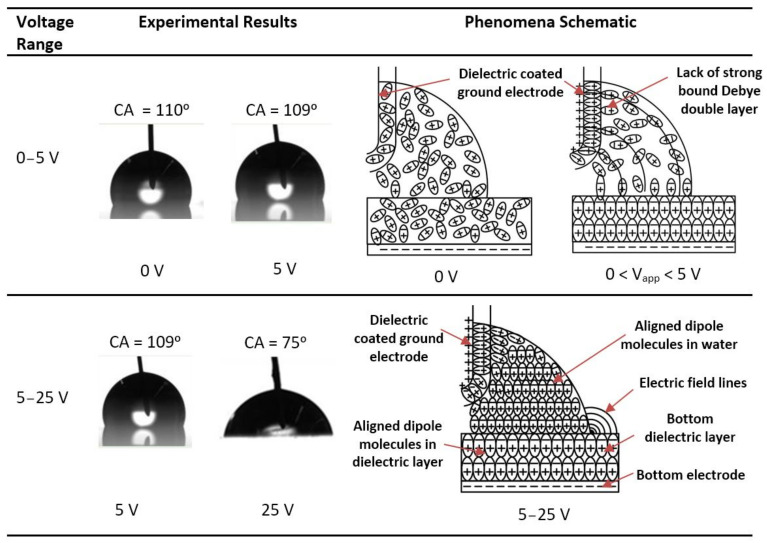
Experimental results and schematic describing the physics of sessile-drop electrowetting with the dielectric-coated ground electrode and increasing DC voltage.

In the electrowetting experiments with a Cytop-coated ground electrode and DC voltage (Figure 4b and Figure 5), the phenomenon of the contact angle increasing (as detected in the electrowetting experiment with the bare ground electrode and DC voltage) was not observed as the voltage increased from zero to the threshold voltage. Two factors define the behaviour of the sessile drop interface on the ground electrode in electrowetting, (a) the macroscopic electromotive force magnitude and direction, (b) the formation of the Debye double-layer. Figure 11 presents the results of the macroscopic electric field simulation considering water to be a leaky dielectric, with a dielectric-coated ground electrode and an applied voltage of 4 V. As seen, a downward electromotive force was exerted to the water interface on the dielectric-coated ground surface. Therefore, no upward force pulls the sessile drop over the ground surface. Although not simulated, this study suggests that the threshold voltage increased on the ground electrode because the metal surface of the ground electrode is covered by a dielectric layer. Therefore, there is a lack of strong bound Debye double-layer electromotive force on top of the dielectric-coated ground electrode, to pull the water droplet on top of the dielectric-coated ground electrode from zero to threshold voltage of electrowetting. This lack of strong bound Debye double-layer on dielectric-coated ground electrode for voltage range 0 V to 5 V (threshold voltage) is shown in the schematic of Figure 15. The sessile drop images at 0 V and 5 V also depict that the contact angle did not increase as in the sessile drop experiment with the bare ground electrode and DC voltage (noted in the previous section). This explanation is also supported by the experimental results, as shown in Figure 5 and Figure 15.

When the dipole molecules of water align themselves with the applied electric field, the water acts as a conducting liquid, and the electric field is concentrated at the triple-phase contact line on the bottom dielectric surface. This is evident from the simulation results shown in Figure 12. Thus, the force on the water adjacent to the bottom electrode spreads the sessile drop and decreases the contact angle. This electric field concentration on the bottom dielectric layer from 5 V to 25 V is also shown in the schematic in Figure 15.

The experimental studies also demonstrate that an exposed bare-ground electrode is not required for electrowetting to occur. In fact, electrowetting also occurs with a dielectric-coated ground electrode. 

### 6.3. Contact-Angle Change in AC Electrowetting with a Bare Ground Electrode

In the experiment with a bare ground electrode and AC voltage, a high frequency (10 kHz) was used to create a steady contact-angle change. To form a stable Debye double-layer, the applied frequency must be lower than the inverse of the charging time of the double-layer [42]. The applied frequency (10 kHz) provides only 0.1 ms, whereas the study [43] suggests that it requires seconds to charge a Debye double-layer. Therefore, given the directional change and high frequency of the applied AC voltage, this study suggests that a stable and strongly bound Debye double-layer may not exist on a bare ground electrode in AC voltage electrowetting. As shown in Figure 5 and Figure 6, the contact angle did not increase as the voltage increased from zero to the threshold voltage (5 V) and decreased from the threshold voltage onwards in this experiment. 

### 6.4. Hysteresis of Contact Angle

In DC electrowetting with a Cytop-coated ground electrode, lower hysteresis was witnessed compared to that in DC and AC electrowetting with a bare ground electrode. In a study by Liu et al. [31], Cytop showed the best sensitivity with respect to contact-angle change with an applied voltage, among various fluoropolymer dielectric materials such as Parylene C, polydimethylsiloxane (PDMS) and self-assembled monolayers (SAMs). Because of the dielectric layer on the ground electrode, there was no upward macroscopic electromotive force on the ground electrode surface; instead, a downward force existed, as seen in the simulation result in Figure 11. Additionally, because of the dielectric layer on top of the ground electrode, this study suggests that the Debye double-layer formation was delayed. Therefore, there was no upward force to contribute to the hysteresis of the contact angle while reducing the applied voltage. As a result, the hysteresis decreased with the Cytop-coated ground electrode in DC electrowetting. This finding provides a new solution to the problem of hysteresis in electrowetting.

Moreover, the hysteresis was lower in AC than in DC electrowetting, in agreement with a previous study [8]. One of the reasons for the hysteresis of contact-angle change in electrowetting is the charge injection into the dielectric layer during the electrowetting process, which would be reduced with an AC voltage.

### 6.5. Comparison of Contact-Angle Changes in AC and DC Electrowetting

Figure 5 reveals that the contact-angle change was lower in AC electrowetting than in DC electrowetting. In DC electrowetting, the charge concentration consistently increases at the triple contact line with increasing voltage, whereas, in AC electrowetting, there is a charge relaxation time due to the alternating character of the AC voltage supply. In this research work, only the positive side of the alternating voltage cycle in AC was used, and the voltage alternated between zero and the applied voltage. Because of this, each cycle had a charge relaxation time when the voltage dropped to zero, which accounts for lower charge concentration. Verheijen and Prins [44] noted the charge relaxation time and associated less charge concentration in AC electrowetting. Hence, the charge concentration was not as high during AC electrowetting as during DC electrowetting, which may account for the lower contact-angle change in AC than in DC electrowetting.

## 7. Conclusions

This study investigated the phenomenon of contact-angle change as the voltage changes from zero to the threshold voltage with a bare ground electrode in DC electrowetting, which, to the authors’ knowledge, had not been observed in prior research. The investigations in this study thoroughly examined the role of the ground electrode in electrowetting and explained the physics of the threshold voltage of electrowetting. Based on the experimental investigation, theoretical explanation, and simulation realisation, the conclusions of this study can be summarised as follows.

A bare ground electrode is not necessary in electrowetting, and electrowetting can also occur with a dielectric-coated ground electrode.

In DC electrowetting with a bare ground electrode, dynamic behaviour is observed as the voltage increases from zero to the threshold voltage that does not follow the Lippmann–Young equation in this range. It is understood that from zero to the threshold voltage, water behaves as a leaky dielectric. From the threshold voltage onwards, it behaves mostly as a conductor and follows the Lippmann–Young equation. As such, Kang’s [17] assumption that the electric field line jumps out from the surface of the sessile drop is valid only beyond the threshold voltage.

The formation of a Debye layer and the leaky-dielectric behaviour of the water drop creates an upward force on the drop on the bare ground electrode as the voltage rises from zero to the threshold voltage in DC electrowetting. However, this phenomenon is not observed in AC electrowetting with a bare ground electrode because the high frequency and alternating direction of AC voltage do not allow a stable Debye double-layer to be formed.

Similarly, with a Cytop-coated ground electrode, this phenomenon is not observed because the Cytop dielectric properties prevent the formation of a strongly bound Debye layer on the ground electrode as the applied voltage increases from zero to the threshold voltage.

For applications where precise control of the contact angle is required, this study suggests that a dielectric-coated ground electrode should be used since it prevents the contact angle from changing dynamically as the voltage rises from zero to the threshold voltage.

The contact-angle change was less in AC than in DC electrowetting. This phenomenon may be related to the lack of a constant charge concentration at the triple contact line in AC electrowetting in contrast to DC electrowetting.

The contact angle hysteresis is lower with a Cytop-coated ground electrode and DC voltage than with a bare ground electrode using AC or DC voltages. Furthermore, the bare ground electrode exhibits less hysteresis in AC than in DC electrowetting. These findings can help researchers solve the contact-angle hysteresis problem in electrowetting applications. 

## Data Availability

There is no external data. All experimental data are contained in the result section.
